# Infective endocarditis due to a novel *Gemella* species revealed by metagenomic sequencing

**DOI:** 10.1128/asmcr.00057-26

**Published:** 2026-08-10

**Authors:** Yoshiyuki Sekikawa, Kazuhiro Horiba, Masanori Hashino, Keiko Soneda, Tomoyuki Kato

**Affiliations:** 1 Department of Infectious Diseases, Japanese Red Cross Musashino Hospital 26830, Tokyo, Japan; 2 Laboratory of Microbial Genomics, Pathogen Genomics Center, National Institute of Infectious Diseases, Japan Institute for Health Security 13511, Tokyo, Japan; 3 Department of Pharmacy, Japanese Red Cross Musashino Hospital 26830, Tokyo, Japan; Vanderbilt University Medical Center, Nashville, Tennessee, USA

**Keywords:** infective endocarditis, metagenomic next-generation sequencing, *Gemella* species, molecular identification, novel bacterial species

## Abstract

**Background:**

*Gemella* species are rare causes of infective endocarditis, and accurate species identification can be challenging with routine diagnostic methods. We report infective endocarditis caused by a novel *Gemella* species, highlighting the diagnostic value of sequencing-based approaches for bacteria not registered in public databases and the risk of misclassification when novel species are not represented in reference databases.

**Case Summary:**

A 55-year-old Japanese man presented with fatigue, anorexia, and altered mental status. Echocardiography revealed vegetations on the mitral and tricuspid valves with severe mitral regurgitation, and imaging demonstrated multiple systemic embolic events. Blood cultures grew gram-positive cocci that were initially identified as *Gemella bergeri* by matrix-assisted laser desorption/ionization time-of-flight mass spectrometry. The patient received antimicrobial therapy and underwent mitral valve replacement and tricuspid valve repair. Culture of the excised valve tissue was negative. Metagenomic sequencing of the valve vegetation detected a *Gemella* species distinct from *G. bergeri*. Genome assembly from sequencing data showed 94.5% average nucleotide identity with a reference *G. bergeri* genome, supporting classification as a novel species. Whole-genome sequencing of the blood culture isolate demonstrated near-complete concordance with the organism detected in the valve tissue. The patient recovered with a 6-week course of intravenous penicillin G after blood cultures became sterile.

**Conclusion:**

This case demonstrates that rare *Gemella* infections may be misidentified by routine mass spectrometry. It underscores the importance of sequencing-based methods for accurate pathogen identification and highlights a diagnostic pitfall associated with database-dependent identification in infective endocarditis.

## INTRODUCTION

Despite advances in diagnostic and therapeutic strategies, infective endocarditis (IE) remains a severe and potentially life-threatening disease with high morbidity and mortality. *Staphylococcus aureus* and viridans group streptococci are the most common causative organisms, whereas *Gemella* species account for fewer than 1% of all IE cases. Because they are difficult to culture, have Gram-variable appearance and biochemical similarities with other species, *Gemella* species are often difficult to distinguish from each other or viridans group streptococci; therefore, molecular identification methods, such as matrix-assisted laser desorption/ionization time-of-flight mass spectrometry (MALDI-TOF MS), are recommended (1). These fastidious gram-positive cocci are difficult to detect using standard blood cultures, and approximately one-third of IE cases caused by *Gemella* are blood culture-negative (2).

*Gemella* species are facultatively anaerobic gram-positive cocci residing in the pharynx, gastrointestinal tract, and genitourinary tract. First identified by Berger in 1961 (3), nine species have since been described (*G. haemolysans*, *G. morbillorum*, *G. bergeri*, *G. sanguinis*, *G. asaccharolytica*, *G. taiwanensis*, *G. parahaemolysans*, *G. palaticanis*, and *G. cuniculi*). Of these, six species (*G. haemolysans*, *G. morbillorum*, *G. bergeri*, *G. sanguinis*, *G. palaticanis*, and *G. cuniculi*) are reported to be pathogenic in humans (4, 5).

Here, we describe a case of IE in a 55-year-old Japanese man in whom the causative organism was initially misidentified as *G. bergeri* by MALDI-TOF MS but was subsequently identified by metagenomic next-generation sequencing (mNGS) to represent a novel *Gemella* species.

## CASE PRESENTATION

A 55-year-old Japanese man presented to the emergency department of a community-based tertiary hospital with a 10-day history of fatigue, anorexia, and disorientation. He was afebrile on admission. His medical history was unremarkable, and he reported no history of recent dental procedures, regular medication use, smoking, alcohol consumption, or illicit drug use.

**Fig 1:**
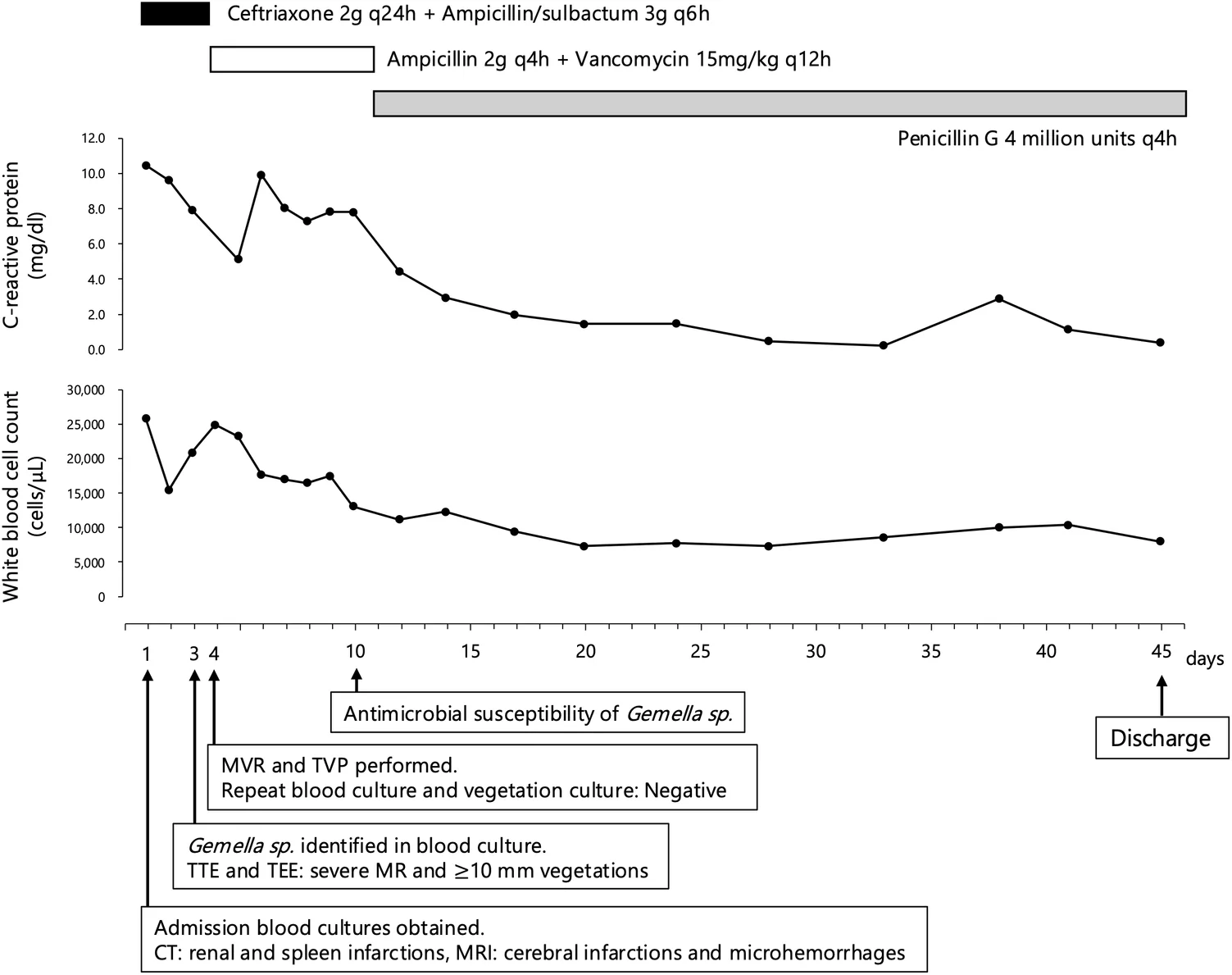
Timeline of key clinical events, diagnostic investigations, therapeutic interventions, and outcomes. The timeline summarizes the patient’s clinical course from the admission through diagnosis and treatment. Major diagnostic evaluations included blood cultures, computed tomography (CT), magnetic resonance imaging (MRI), transthoracic echocardiography (TTE), transesophageal echocardiography (TEE), and metagenomic next-generation sequencing (mNGS).

**Fig 2:**
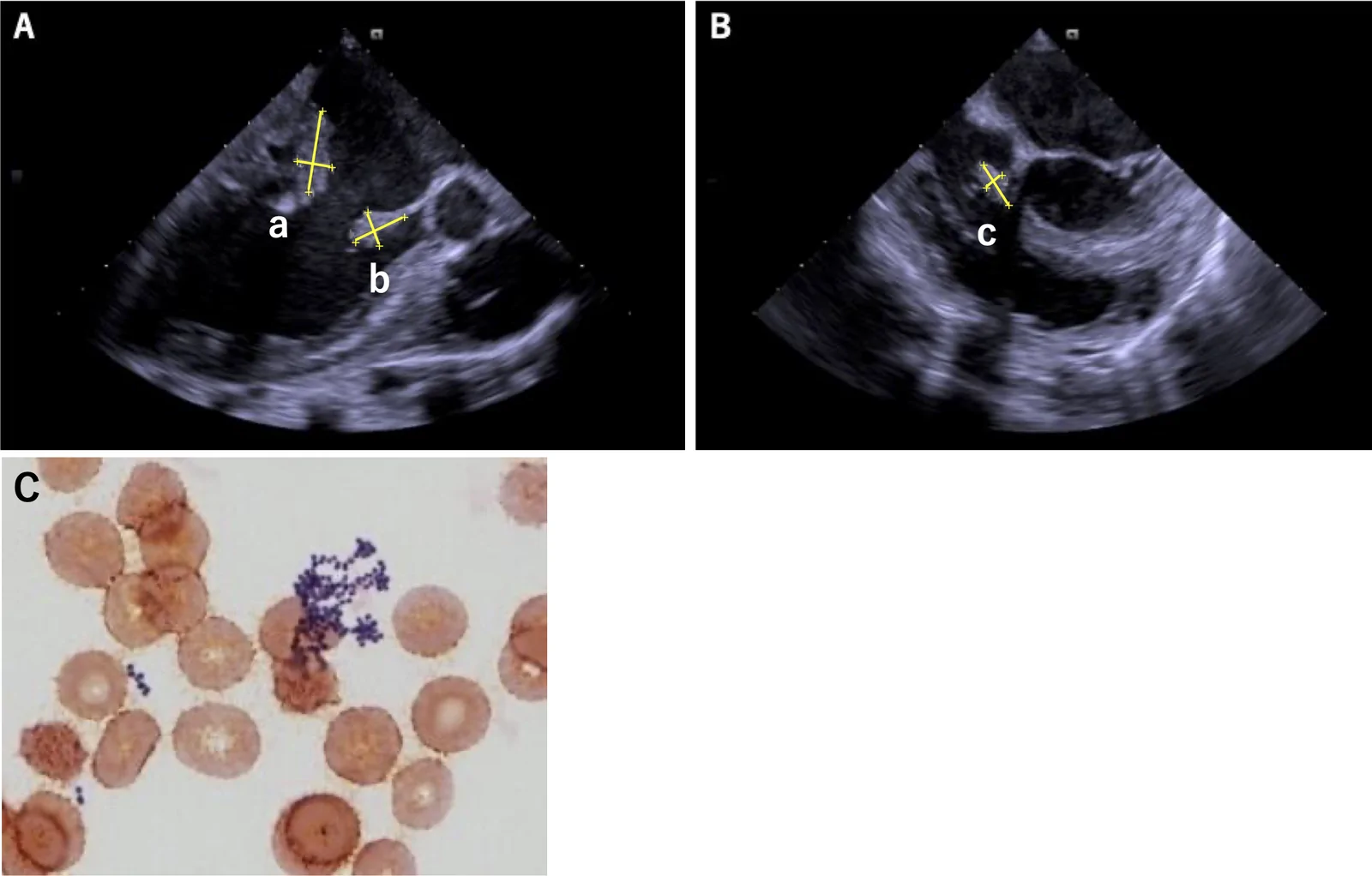
Clinical imaging and microbiological findings of the patient with infective endocarditis. (**A**) Transesophageal echocardiography demonstrated a flail posterior leaflet (P2 segment) with ruptured chordae produced an eccentric mitral regurgitation jet directed toward the anterior left atrium. Large vegetations on both the posterior (a; 21.4 × 9.6 mm) and anterior (b; 15.3 × 8.9 mm) mitral valve leaflets. (**B**) Vegetation (c; 11.6 × 6.4 mm) on the tricuspid valve. (**C**) Gram stain of the positive blood culture showed gram-positive cocci arranged in short chains and small clusters (×1,000).

On arrival, his vital signs were as follows: temperature, 37.0°C; blood pressure, 104/70 mm Hg; heart rate, 98 beats/min (regular); respiratory rate, 16/min; and peripheral capillary oxygen saturation (SpO_2_), 98% on room air. Physical examination revealed petechiae on the palpebral conjunctiva and soft palate. No Osler nodes or Janeway lesions were observed. A holosystolic murmur (Levine grade III/VI) was audible at the cardiac apex. Laboratory findings showed leukocytosis (white blood cell count, 25,900/μL [reference range, 3,300–8,600/μL]), elevated C-reactive protein (10.48 mg/dL [reference range, ≤0.14 mg/dL]), and serum creatinine of 0.76 mg/dL (reference range, 0.65–1.07 mg/dL). HbA1c was 5.9% (reference range, 4.9–6.0%), and HIV antigen/antibody testing was negative. Electrocardiography and chest radiography were unremarkable. Based on the patient’s symptoms and physical findings, infective endocarditis was strongly suspected, and computed tomography (CT), magnetic resonance imaging (MRI), and transthoracic echocardiography were performed on the same day. Abdomen and pelvis CT revealed bilateral renal and splenic infarctions. Brain CT and MRI demonstrated multiple acute cerebral infarctions and subcortical and subarachnoid microhemorrhages without evidence of cerebral aneurysm. Transthoracic echocardiography revealed severe mitral regurgitation with 10 mm vegetations on both the anterior and posterior mitral valve leaflets. Transesophageal echocardiography confirmed vegetations on the mitral and tricuspid valves (Fig. 1 and 2).

Empiric antimicrobial therapy with ceftriaxone (2 g every 24 h) and ampicillin/sulbactam (3 g every 6 h) was initiated by a cardiologist upon admission in accordance with the 2017 Japanese guidelines for infective endocarditis (6). On hospital day 3, three sets of blood cultures obtained using the BD BACTEC System (Becton, Dickinson and Company, Franklin Lakes, NJ, USA) yielded gram-positive cocci (Fig. 2C). MALDI Biotyper (Bruker Daltonics, Bremen, Germany) identified the isolate as *Gemella bergeri* (score value: 2.03). The patient was diagnosed with definite IE. Pending antimicrobial susceptibility results, antimicrobial therapy was modified to ampicillin (2 g every 4 h) plus vancomycin (15 mg/kg every 12 h). Dental evaluation by a dentist revealed chronic periodontitis and caries with pulp involvement of the right maxillary second molar. On hospital day 4, the patient underwent mitral valve replacement and tricuspid valve repair using a pericardial patch. Surgery was performed because multiple vegetations larger than 10 mm were identified on the mitral and tricuspid valves; rupture of the chordae tendineae of the posterior mitral leaflet had resulted in severe mitral regurgitation; and the patient showed signs of progressive heart failure, including a small pleural effusion and elevated brain natriuretic peptide (BNP) levels (388 pg/mL; reference range, ≤18.4 pg/mL). Although brain MRI demonstrated subcortical and subarachnoid microhemorrhages, no focal neurological deficits were observed, and early surgery was, therefore, considered feasible. Blood cultures obtained on the day of surgery were negative, and valve tissue cultures were also negative. On hospital day 10, therapy was de-escalated to intravenous penicillin G (4 million units every 4 h) after antimicrobial susceptibility testing confirmed the susceptibility of *G. bergeri* to penicillin G. The postoperative course was uneventful, and penicillin G was continued for 6 weeks after blood cultures became sterile. Neurocognitive function gradually improved during treatment, as evidenced by neuropsychological testing. The Mini-Mental State Examination score improved from 15/30 on hospital day 10 to 25/30 on hospital day 45. Raven’s Colored Progressive Matrices testing was limited to Set A on hospital day 11 because of the patient’s physical condition, with a score of 5/12, whereas full sets A–C were completed on hospital day 41, yielding a score of 30/36.

The mNGS of the valve vegetation was performed at the National Institute of Infectious Diseases using previously described methods based on direct shotgun metagenomic sequencing (7). *De novo* assembly of the mNGS data yielded a draft genome of *Gemella* sp., which showed an average nucleotide identity (ANI) of 94.5% with a *G. bergeri* (GCA_000469465.1). This genome was registered in GenBank as a novel Gemella species, *Gemella* sp*.* Musashino-2025 (GCF_054771455.1). Whole-genome sequencing (WGS) of the isolate from blood cultures, which was initially misidentified as *G. bergeri* by the MALDI Biotyper, revealed that its genome also shared 99.9957% ANI with the draft genome assembled from the mNGS data, confirming that both represented the same *Gemella* species.

## DISCUSSION

*Gemella* species can occasionally act as opportunistic pathogens, causing not only IE but also septic arthritis, osteomyelitis, vertebral osteomyelitis, empyema, lung abscess, and meningitis (8). A review of 52 reported cases of infective endocarditis (IE) caused by *Gemella* species indicated that *G. morbillorum* (46.3%) and *G. haemolysans* (25.9%) were the most frequently identified pathogens. Most patients were male (79.6%) and presented with a wide range of symptoms, including fever, malaise, fatigue, myalgia, and dyspnea. Underlying conditions commonly reported included recent dental procedures, congenital heart disease, prosthetic valves, and immunocompromised states (9). In the present case, the patient also presented with nonspecific symptoms, including fatigue, anorexia, and malaise, and was noted to have dental caries. Because they are difficult to culture, have Gram-variable appearance, and biochemical similarities with other species, *Gemella* species are often difficult to distinguish from each other or viridans group streptococci. In our case, the causative organism was initially difficult to classify accurately, highlighting the diagnostic challenges associated with *Gemella* infections. These microbiological characteristics, combined with nonspecific clinical features, may contribute to delayed diagnosis.

Accurate identification of *Gemella* species is also important for appropriate antimicrobial selection. *Gemella* species are typically susceptible to penicillin G, ampicillin, and ceftriaxone. Gentamicin is frequently co-administered to achieve synergistic effects (10). The treatment durations of 4–6 weeks were reported without species-specific differences in clinical outcomes. Prognosis is generally favorable when appropriate antimicrobial therapy is administered (9, 11). In our case, penicillin G monotherapy was administered for 6 weeks without gentamicin in accordance with the 2015 American Heart Association guidelines for IE (10) after confirmation of antimicrobial susceptibility.

In a case series of IE caused by *Gemella* species, blood cultures were frequently negative, and 31% of cases were diagnosed solely by 16S rRNA PCR performed on excised valve tissue (2). These findings show the critical importance of molecular methods for the accurate identification of *Gemella* species.

*G. bergeri* was first reported in 1998 (12), and eight cases of IE due to this species have been described (8, 12–16) (Table 1). Previous cases were identified using conventional culture-based or molecular methods, and none of these eight reported cases used MALDI-TOF MS for species identification (Table 1). The present case highlights the potential value of genomic analyses for confirming species-level identification within the genus *Gemella*. WGS and ANI analyses provide a robust framework for bacterial species delineation, with ≥95% ANI generally regarded as the threshold for species definition (17). The 94.5% ANI observed in this case supports the classification of the bacteria as a novel species.

**TABLE 1:** Reported cases of infective endocarditis caused by *Gemella bergeri^a^*

Case	Age (yr)	Sex	Underlying conditions	Culture (specimen)	Antibiotics	Operation	Duration (weeks)	Outcome	Reference
1	15	Male	Tetralogy of Fallot, pulmonary atresia	Blood	VAN + GEN	NA	6	Survived	(8)
2	42	Male	Mitral valve prolapse	Blood	NA	Mitral valve replacement	NA	Survived	(12)
3	NA	NA	NA	Blood	NA	NA	NA	Survived	(12)
4	NA	NA	NA	Blood	NA	NA	NA	Survived	(12)
5	50	Male	Bicuspid aortic valve	Blood	CTX + GEN	NA	4	Survived	(13)
6	32	Male	Bicuspid aortic valve, asthma, dyslipidemia, gastroesophageal reflux disease	Blood, valve	AMP + GEN	Aortic valve replacement	NA	NA	(14)
7	37	Male	Tricuspid valve cleft	Valve	NA	Tricuspid valve replacement	4	Survived	(15)
8	24	Male	Rheumatic heart disease	Blood	CTX + GEN	NA	NA	Died	(16)

^*a*^ AMP, ampicillin; CTX, ceftriaxone; GEN, gentamicin; NA, not available; VAN, vancomycin. Outcome: “Survived” indicates the patient was alive at discharge, while “Died” indicates death during hospitalization.

Our case highlights the limitations of relying solely on MALDI-TOF MS and emphasizes the importance of integrating sequencing-based approaches to resolve diagnostic uncertainty and improve pathogen characterization. Accurate species identification has implications not only for taxonomy but also for epidemiological understanding and potentially for optimization of antimicrobial strategies in rare pathogens. Although conventional microbiological methods, including MALDI-TOF MS and antimicrobial susceptibility testing, were sufficient for clinical management in the present case, advanced sequencing technologies may provide added value in selected situations. Given their costs, turnaround times, and limited availability, routine implementation for all cases of infective endocarditis is not currently practical. Instead, mNGS and WGS may be particularly useful for culture-negative endocarditis, discrepant microbiological findings, unusual pathogens, outbreak investigations, or cases requiring detailed epidemiological and taxonomic characterization.

## Data Availability

The full data supporting the findings of this study are available from the corresponding author upon reasonable request. Sequencing reads derived from the cultured bacteria reported in this case study were deposited in the DDBJ Sequence Read Archive under experiment numbers DRX769939–DRX769940.
